# Minimally invasive surgery in neurogenic tumours

**DOI:** 10.3332/ecancer.2031

**Published:** 2025-11-13

**Authors:** Samer Michael, J Ted Gerstle

**Affiliations:** 1 Department of Paediatric Surgery, Hadassah University Medical Center, Kiryat Hadassah, Jerusalem, Israel; 2 Paediatric Surgery Service, Memorial Sloan Kettering Cancer Center, 1275 York Avenue, New York, NY, USA; 3 Faculty of Medicine, The Hebrew University of Jerusalem, Jerusalem, Israel

**Keywords:** neurogenic, neuroblastoma, minimally invasive surgery

## Abstract

The use of minimally invasive surgery (MIS) for the diagnosis and treatment of neurogenic tumours has markedly increased over the past decade, evolving from a diagnostic and staging tool to a therapeutic option in carefully selected cases. The advantages of MIS—reduced postoperative pain, shorter hospital stay, and faster recovery—must be weighed against its technical challenges, including limited operative space, loss of tactile feedback and increased risk when image-defined risk factors are present. This chapter reviews current evidence, outlines practical indications and contraindications and proposes structured guidelines for MIS in paediatric neurogenic tumours to assist surgeons in safe adoption while maintaining oncologic integrity.

## Introduction

The use of Minimally Invasive Surgery (MIS) for the diagnosis and treatment of neurogenic tumors has markedly increased over the past decade, ranging from biopsy and staging to definitive resection. [1, 2].

Although there are no prospective studies nor randomised trials [3, 4], several retrospective studies and systematic reviews were conducted to compare the safety and outcomes of MIS and open surgery.

MIS in paediatric surgery in general and in oncologic surgery in particular has unique challenges. Patients have smaller bodies, restricting space for large tumours, the need for a larger incision for extraction, difficulty in navigation or limited room for stapler, use of adequate instrumentation and anaesthesia difficulties. Additionally, due to the lower number of cases, it is more difficult to gain experience and the learning curve might be an issue [5]. Moreover, the larger size and vascular encasement in neurogenic tumours pose an additional challenge for MIS.

### Advantages and disadvantages of MIS

The advantages of MIS under the correct indications are similar to other fields and include reduced postoperative pain, lower incidence of ileus, shorter hospital stays and earlier return to activity [1].

Although not statistically significant, Phelps *et al* [6] found a trend toward earlier initiation of adjuvant chemotherapy after MIS resection of embryonal tumours, including neuroblastoma.

The use of MIS for tumour biopsy is supported by several studies to be safe and feasible [7–13].

Despite the potential advantages of MIS, neurogenic tumours are technically and ontologically more challenging to resect than other diseases. From a technical standpoint, the tumours are often large and difficult to mobilise in a small space with reduced visibility and lacking tactile feedback. In addition, the ability to control injury to encased vessels is reduced [1, 6].

From an oncological standpoint, the ability to achieve complete macroscopic resection in locally advanced tumours is difficult, risky and requires exceptional MIS skills and experience.

### Feasibility, safety and outcomes

In a study of oncologic integrity and safety of MIS versus open surgery [6], 101 patients (50%) had neuroblastoma, of which, 20 patients underwent MIS.

MIS was correlated with older age, greater body surface area, stage I and stage II tumours, smaller tumour volume and no image-defined risk factors (IDRFs).

Almost all patients undergoing MIS resection had ≥98% tumour resection based on measurement of residual tumour on postoperative imaging, which may be attributed to the lower number of IDRFs and the greater proportion of upfront resections in this group

Relapse-free survival and overall survival (OS) did not differ significantly between the two approaches after stratification by stage and risk category.

In a study of the National Cancer Data Base from 2010 to 2012 [9], 17% (98 of 579) of children with neuroblastoma underwent MIS: patients were more likely to have a thoracic tumor, smaller size, and no metastatic disease.

After propensity score matching, there were 196 children undergoing open surgery compared with 98 children undergoing MIS. There was no difference between open and MIS surgery for 30-day mortality, readmissions, surgical margin status and 1- and 3-year survival. Median hospital length of stay was shorter in MIS versus open surgery (3 versus 4 days; *p* < 0.01). Among MIS cases, 7% were robotic assisted and 12.2% were converted to open.

In 2022, A SIOPEN multicentre study [12] reviewed 222 cases of MIS resection for neurogenic tumours. 54% were adrenal lesions, followed by 35% thoracic tumours. 73.4% had no IDRFs. A conventional laparoscopic approach was used in the majority of abdominal and pelvic tumours, 11 patients had retroperitoneoscopic resection and 1 had robotic surgery.

The number of ports for both thoracoscopic and laparoscopic procedures was 3–4, with no reported port site recurrences. The median duration for surgery was 120 minutes (range = 40–530 minutes). Resection of lymph nodes, in addition to the primary, was performed in 32 patients (14.4%).

The surgery was converted to open in 23 patients (10%) due to significant scarring, fibrosis, invasion of renal pedicle or tumour encasement of major vessels in the chest or the abdomen.

Reported complications included significant blood loss, Horner's syndrome, chylothorax and renal atrophy. The overall 30-day postoperative complication rate for all patients was 10% (*n* = 21) (9 Grade I, 6 Grade II and 6 Grade III according to Clavien-Dindo grading system).

Univariate analysis showed that thoracic procedures, presence of IDRF and tumour volume >60 mL were the main significant risk factors for open conversion.

Reasons for open conversion notably include close proximity of tumour to major blood vessels, intercostal disease infiltration and significant intra operative bleeding.

Volume >75 mL was significantly associated with complications. The tumour volume, however, did not modify the extent of resection or the recurrence rate.

Thoracic procedures were found to be associated with a higher rate of complications and increased risk of incomplete macroscopic resection compared to abdominal operations.

L2 staging and consecutively the presence of one or more than one IDRF were statistically significant factors for conversion to open surgery, incomplete resection, development of complications and recurrence of the tumour.

On further multivariate analysis, the presence of more than one IDRF remained a statistically significant risk to conversion to open. L2 stage remained a risk for complications but not for conversion, incomplete resection or recurrence. Tumour volume above 100 mL was a risk factor for conversion to open and development of complications, while MYCN amplification was a risk factor for incomplete resection.

### Indications and contraindications

MIS is typically used in smaller volume and earlier stage neuroblastic tumours without IDRFs [6, 7, 14, 15]. The emphasis on patient selection according to size and IDRFs is supported by several studies. Laparoscopic resection of abdominal and adrenal neuroblastomas was performed on tumours ≤60 mm in diameter with no IDRFs, with a low complication rate and outcomes comparable to open surgery [7, 15].

The surgeon should also consider the subtotal encasement and deformation of major vessels as a contraindication to MIS [16].

In a systematic review by the American Paediatric Surgical Association Cancer committee in 2020 [4], the authors concluded that abdominal MIS is feasible and can be safely performed for carefully selected tumours with acceptable morbidity. Conclusions could not be drawn regarding disease-free or OS outcomes. The absence of IDRFs appears to be a safe criterion for the utility of MIS. Complications and conversion to open procedures are more common in the presence of IDRFs, and for these patients, open resection may be preferable. They also concluded that optimal tumour size ranges from 4 to 6 cm without IDRF's, with a weak evidence level.

Regarding thoracic MIS, they concluded that it seems feasible, safe and effective. With reported complications comparable to thoracotomy. Thoracic neurogenic tumours may be more amenable to MIS resection in the absence of IDRF's.

Although the size of the primary tumour as a criterion for MIS resection of thoracic neuroblastic tumours has not been as extensively studied, in their review, they found that tumour size ranged between 2  and 18 cm and the most commonly reported sizes were between 3 and 5 cm.

They found comparable rates of complications compared to the open approach and are likely more attributable to tumour location rather than method of resection. However, it should be noted that tumours undergoing MIS resection in the reported studies were highly selected, which may explain improved morbidity.

The authors emphasised that the use of MIS techniques on inappropriate patients or by surgeons with limited MIS skills could have detrimental outcomes. Patients should be highly selected, mainly based on size and the presence of IDRF's.

In 2010, The International Paediatric Endosurgery Group published guidelines (class III evidence) for the surgical management of adrenal masses in children [17].

The consensus suggested that for advanced neuroblastoma, laparoscopic biopsy may be performed, and that laparoscopic resection of small, localised tumours may be performed as long as the principles of oncologic surgery are maintained. MIS can also be considered for higher risk disease if the tumour responds favourably to neoadjuvant chemotherapy. A strong recommendation was made by this group to remove all tumours using an endoscopic retrieval bag.

The use of ICG during MIS for neuroblastic tumours has been described; however, further research is required to establish its benefits [18].

In our experience, the use of a number of IDRFs to predict surgical complexity is limited and should be used with caution. Alternatively, we recommend using the following terms and definitions to describe the relationship of the tumour to any adjacent critical structure [19]:

- Contact: no visible layer is present between the tumour and adjacent structure <50% of the vessel's circumference (not an IDRF).
- The term flattened is used to describe veins with reduced diameter that still have a partially visible lumen (not an IDRF).
- Encasement for a vessel means >50% of the vessel circumference is in contact with tumour (IDRF).
- Total encasement means that a vital structure is completely surrounded by tumour (IDRF).
- A flattened vein with no visible lumen is considered to be encased (IDRF).
- Compression is used only when referring to airways. When tumour contacts an airway and causes the short axis to be reduced, this is considered an IDRF.
- For other vital structures, a contact may cause displacement (abnormal anatomic location) or distortion (abnormal anatomic shape) (not IDRF).

In addition to the circumference of contact with the vessel, the length of contact should also be noted, for longer contact increases the risk for vessel injury, which is more difficult to manage in MIS.

In retroperitoneal and pelvic tumours, the contact and encasement of the ureter should also be taken into consideration in the decision on MIS suitability.

We highly recommend using intraoperative neurophysiological monitoring (IONM) in thoracic tumours with involvement of intervertebral foramina or tumour with proximity to pelvic nerves.

A summary of suggested guidelines and practical recommendations for patient selection and surgical approach is provided in Table 1.

### Robotic-asssisted MIS (RA-MIS)

Although it potentially has technical advantages in precision and maneuvering, it is not surprising that robotic MIS faces more challenges in paediatric oncology. A multicentre review of RA-MIS in paediatric cancer included 22 thoracic and abdominal neuroblastic tumours [20]. They showed that it is technically feasible and safe to perform in select cases. Providing the benefits of MIS, such as reduced length of stay and improved cosmesis, robotic-assisted surgery also provides the added benefits of improved dexterity and enhanced visualisation, including three-dimensionality.

The high-volume Paediatric Robotic Program at Hôpital Necker-Enfants malades (Paris) reported robotic resection of 51 neurogenic tumours in 47 children; 57% adrenal and 19% posterior mediastinum. 6 (11%) patients had vascular encasement (3 renal pedicle, 2 iliac vessel and 1 vena cava encasement).

They reported one conversion and no emergency undocking. Resection was macroscopically complete in all cases (confirmed on post-operative imaging).

There were no cases of vascular injury or post-operative complications [10].

It is important to note that these results should be interpreted cautiously, since the surgeries were performed in a high-volume paediatric robotic surgery centre and 89% of patients had no IDRFs.

### Surgical approach

#### Thoracoscopic resection

##### 1. Patient positioning

- Use an appropriate size, use a bean bag or other supportive devices to stabilise the patient.
- Patient needs to lie on foam or gel pads to minimise pressure injury risks.
- Place the patient in a 45-degree semi-prone position with the tumour side up, for gravity to drop the lungs away from the spine.
- The operating table should be slightly flexed to widen the intercostal spaces and improve thoracic access.
- In younger patients, the beanbag can be used for flexion.
- The patient should be as close as possible to the edge of the table towards the operator.
- The operator should stand anterior to the patient.

Arms are positioned to allow access without obstruction while protecting from neuropraxia [9–13].

##### 2. Trocar sites

- Use a triangulation approach for optimal visualisation and instrument maneuverability:
  1. Camera port:
    - Typically placed in the midaxillary line in the 5th or 6th intercostal space.
  2. Working port 1:
    - Typically placed 2–3 cm anterior to the midaxillary line, in the 4th or 5th intercostal space.
  3. Working port 2:
    - Placed posterior to the midaxillary line above the costophrenic junction (to allow enough length for the stapler).
- Ports should be placed under vision to avoid damage to major vessels, lungs or diaphragm.
- Additional ports (should be used as needed).

##### 3. Surgical technique

- Use a lower insufflation pressure (6–8 mmHg) to minimise cardiovascular and respiratory issues.
- Tumour dissection should proceed from the peripheral to the central area and saving division of critical structures until the tumour has been isolated.
- Identify critical structures to avoid injury.
- Use Endo Catch bag for tumour retrieval.

##### 4. Tips and pitfalls

Tips:

- Memorise preoperative imaging and relative location of vascular and other vital structures.
- Display imaging during procedure for frequent reference.
- Go over the rescue plan for an intraoperative bleeding with the team before the start of procedure.
- Patient safety and oncologic principles should be prioritised with a low threshold to convert to open surgery.
- We highly recommend using IONM in thoracic tumours with involvement of intervertebral foramina.

Pitfalls:

- The use of MIS should not compromise the oncologic principles of resection, sampling and reporting of operative findings, according to other most recent oncology groups' guidelines.
- Resection extent should not be sacrificed in favour of a minimally invasive approach.
- Inappropriate patient selection.
- OR team members, including anaesthesia and surgery teams not experienced with paediatric MIS.
- Not positioning patient appropriately.
- Tissue planes are difficult after neoadjuvant therapy.
- Not recognising feeding and draining vessels.

#### Laparoscopic adrenalectomy/retroperitoneal tumour resection

##### 1. Patient position

- Place the patient in a lateral decubitus position, with the tumour side up.
- Use an appropriate size, use a bean bag or other supportive devices to stabilise the patient.
- The operating table should be slightly flexed at the lumbar region to open up the retroperitoneal space and improve access.
- Patient needs to lie on foam or gel pads to minimise pressure injury risks.
- In younger patients, the beanbag can be used for flexion.
- The patient should be as close as possible to the edge of the table towards the operator.
- The operator should stand anterior to the patient.
- Arms are positioned to allow access without obstruction while protecting from neuropraxia.

##### 2. Trocar sites

- Use a triangulation approach for optimal visualisation and instrument maneuverability:
  1. Camera port:
    - Typically placed at the umbilicus.
  2. Working port 1:
    - Mid-clavicular line, about 2–4 cm below the costal margin.
  3. Working port 2:
    - Anterior axillary line, about 2–4 cm below the costal margin.
  4. Accessory port (optional):
    - Positioned in the epigastric region or lower quadrant, as needed, for retraction or stapling.

##### 3. Surgical technique

- Maintain a low insufflation pressure (8–10 mmHg) to avoid compromising ventilation or venous return in small paediatric patients.
- Mobilise the tumour or adrenal gland working medially from the lateral border.
- Identify critical structures to avoid injury.
- Identify and isolate the adrenal vein early in the procedure.
- For the right adrenal gland, locate the adrenal vein draining into the inferior vena cava.
- For the left adrenal gland, identify the vein draining into the left renal vein.
- Secure the adrenal vein with clips or an energy-sealing device before transection.
- Use Endo Catch bag for tumour retrieval.

##### 4. Tips and pitfalls

Tips:

- Memorise preoperative imaging and relative location of vascular and other vital structures.
- Display imaging during procedure for frequent reference.
- Go over the rescue plan for an intraoperative bleeding with the team before the start of procedure.
- Patient safety and oncologic principles should be prioritised with low threshold to convert to open surgery.

Pitfalls:

- The use of MIS should not compromise the oncologic principles of resection, sampling and reporting of operative findings, according to other most recent oncology groups guidelines.
- Resection extent should not be sacrificed in favour of a minimally invasive approach.
- Inappropriate patient selection.
- OR team members including anaesthesia and surgery teams not experienced with paediatric MIS.
- Not positioning the patient appropriately.
- Tissue planes are difficult after neoadjuvant therapy.
- Not recognising feeding and draining vessels.
- Secure vessels early to prevent uncontrolled bleeding.
- Adrenal vein misidentification: Mistaking the adrenal vein for surrounding structures (e.g., renal vein or inferior vena cava) can lead to complications.
- Instrument crowding: Paediatric patients’ small abdominal cavities can limit space for instruments; plan trocar placement carefully.

#### Laparoscopic pelvic tumour resection

##### 1. Patient position

- Patient in supine position.
- Elevate the pelvis slightly using a gel roll to improve access to the presacral space.
- Adjust the table to a Trendelenburg tilt to allow the bowel to fall cranially, enhancing visualisation of the pelvis.
- Arms are secured and padded to prevent nerve injury.

##### 2. Trocar sites

- Use a triangulation approach to provide optimal visualisation and maneuverability:
  - Camera port:
    Positioned at or just above the umbilicus.
  - Working port 1:
    Placed in the right lower quadrant, at the midclavicular line, about 2–3 cm above the anterior superior iliac spine.
  - Working port 2:
    Placed in the left lower quadrant, symmetric to the first working port.
  - Accessory port (optional):
    Positioned in the suprapubic region for retraction or additional instrumentation, if necessary.

##### 3. Surgical technique

1. Maintain a low insufflation pressure (8–10 mmHg) to avoid compromising ventilation or venous return in small paediatric patients.
2. Pelvic neurogenic tumours should have a clear plane separating them from the rectum and colon mesentery, which should be followed for mobilisation.
3. The ureter is usually pushed away by the tumour and should be dissected off bluntly with caution.
4. Identify critical structures such as the sacral plexus, iliac vessels and ureters to avoid injury.
5. Place the tumour in an Endo Catch bag for extraction through the largest port or a mini-incision.

##### 4. Tips and pitfalls

Tips:

- Memorise preoperative imaging and relative location of vascular and other vital structures.
- Display imaging during procedure for frequent reference.
- Go over the rescue plan for an intraoperative bleeding with the team before the start of procedure.
- Patient safety and oncologic principles should be prioritised with low threshold to convert to open surgery.
- Ensure the urinary bladder and rectum are empty before surgery to reduce risk of injury and improve visualisation.
- We highly recommend using IONM in tumours with proximity to pelvic nerves.

Pitfalls:

- The use of MIS should not compromise the oncologic principles of resection, sampling and reporting of operative findings, according to other most recent oncology groups guidelines.
- Resection extent should not be sacrificed in favour of a minimally invasive approach.
- Inappropriate patient selection.
- OR team members, including anaesthesia and surgery teams not experienced with paediatric MIS.
- Not positioning patient appropriately.
- Tissue planes are difficult after neoadjuvant therapy.
- Not recognising feeding and draining vessels.
- Secure vessels early to prevent uncontrolled bleeding.
- Instrument crowding: Paediatric patients’ small abdominal cavities can limit space for instruments; plan trocar placement carefully.

## Conclusion

MIS is a safe and effective option for selected paediatric neurogenic tumours, particularly smaller, localised cases without IDRFs. While it offers benefits like reduced pain and faster recovery, its success depends on careful patient selection, adherence to oncologic principles and surgical expertise. MIS should only be attempted in appropriate cases, with a low threshold for conversion to open surgery to ensure patient safety and optimal outcomes. Further research is needed to strengthen evidence and refine guidelines.

## List of abbreviations

IDRFs, Image-defined risk factors; IONM, Intra-operative neural monitoring; MIS, Minimally invasive surgery; RA-MIS, Robotic-assisted MIS; SMA, Superior mesenteric artery; SVC, Superior vena cava.

## Conflicts of interest

The authors declare that they have no conflicts of interest.

## Funding

The authors declare that they have no financial conflicts of interest.

## Figures and Tables

**Table 1.** Suggested Guidelines and Recommendations

	May consider MIS	Relative contraindications	Absolute contraindications	Notes
All sites	Diagnostic biopsy Small, localised tumour without IDRFs Upfront resection without neoadjuvant chemotherapy	Contact with large vessel Involvement of intervertebral foramina Tumour volume 60–75 mL s/p Neoadjuvant chemotherapy	One or more IDRF Significant Contact (length, % circumference flattening) with large vessel Intra spinal involvement >75 mL Advanced stage neuroblastoma Invasion to adjacent organ Tumour occupying most of the cavity or causing significant displacement or distortion of adjacent structures. Tumour size in relation to patient size expected to have limiting space impairing proper visualisation and safe dissection Lesion is a metastatic node	Consider observation only when clinically indicated. Multidisciplinary tumour board discussion is strongly advised to agree on suitability of MIS. Use Endo Catch bag for extraction in tumour resections. The use of MIS should not undermine adherence to oncologic principles of resection and reporting of operative findings. according to other most recent oncology groups guidelines The rescue plan for an intraoperative bleeding should be prepared by the operating surgeon. Low threshold to conversion to open surgery. Evaluate the risk of any anaesthetic complications caused by mass effect together with the anaesthesiology team prior to deciding the optimal procedure, which must be individualised to each clinical scenario.
Thoracic	Paraspinal tumour Tumour <6 cm or 60 mL		Involving thoracic inlet or apex Significant contact with aorta, subclavian vessels, superior vena cava, main bronchus, trachea, esophagus and pericardium Tumour crossing midline. Mass is more than one third of the thorax. Left sided tumour located in T9–T12 (risk for the Adamkiewicz artery)	Single lung ventilation is often required to provide adequate operating space
Adrenal	Small, localised tumour ‘simple adrenalectomy’		Suspected adrenocortical carcinoma Tumour crossing midline. Pancreatic involvement Significant contact with IVC, renal pedicle, aorta, porta hepatis, superior mesenteric artery (SMA) and Celiac trunk Lymph node involvement	Either transperitoneal or retroperitoneal laparoscopy can be employed depending on surgeon’s experience
Abdominal non-adrenal	Small, localised tumour		Significant contact with IVC, renal pedicle, aorta, porta hepatis, SMA and celiac trunk Lymph node involvement Tumour crossing midline. Any renal hilum or pancreatic involvement Significant contact with the ureter	Either transperitoneal or retroperitoneal laparoscopy can be employed depending on surgeon’s experience
Pelvis	Pre sacral		Extension into intervertebral foramina Significant contact with Iliac vessels or nerve plexus Significant contact with the ureter	IONM recommended in tumours with lateral extension
